# VeSpAR trial: a randomized controlled trial comparing vessel-sparing anastomotic repair and transecting anastomotic repair in isolated short bulbar urethral strictures

**DOI:** 10.1186/s13063-020-04712-5

**Published:** 2020-09-11

**Authors:** Wesley Verla, Marjan Waterloos, Mieke Waterschoot, Benjamin Van Parys, Anne-Françoise Spinoit, Nicolaas Lumen

**Affiliations:** Department of Urology, Ghent University Hospital, C. Heymanslaan 10, 9000 Ghent, Belgium

**Keywords:** Urethral stricture, Urethroplasty, Anastomotic repair, End-to-end, Excision and primary anastomosis, Vessel-sparing, Non-transecting, Transecting, Randomized controlled trial

## Abstract

**Background:**

Vessel-sparing anastomotic repair (vsAR) has been developed as a less traumatic alternative to transecting anastomotic repair (tAR) to treat isolated short bulbar urethral strictures. This vessel-sparing technique could result in improved functional outcomes without jeopardizing the excellent surgical outcome after (transecting) anastomotic repair. The purpose of this study is to directly compare vsAR and tAR for both surgical and functional outcomes.

**Methods:**

This trial is a prospective, interventional, multi-center, single-blinded, 1:1 randomized, controlled, non-inferiority, phase II trial. Sample size calculation resulted in a required sample size of 100 patients (50 patients per arm). Trial participants will be randomized by an independent third party using a computer-based random sequence generator with permuted blocks of variable size. The primary objective of this trial is to show that vsAR is non-inferior to tAR in terms of failure-free survival after 24 months of follow-up, considering a non-inferiority limit of 10%. Failure is defined as the inability to pass a 16-Fr flexible cystoscope through the reconstructed area without damaging the urethral mucosa. Secondary end-points mainly include differences in postoperative complications and changes in functional outcome parameters, which will be assessed with validated questionnaires. All participants are scheduled for follow-up at 3, 12, and 24 months postoperatively.

**Discussion:**

This trial will provide level Ib evidence about the differences in both surgical and functional outcome between vsAR and tAR, which may importantly scape the future of bulbar urethral reconstruction. Depending on the trial results, this phase II trial may generate a larger phase III trial with more statistical power and a lower alpha value.

**Trial registration:**

This trial is registered at clinicaltrials.gov (NCT03572348) and in the Belgian Clinical Trial Registry (B670201837335). The trial was registered prospectively. Registered on 28 June 2018.

## Administrative information

Note: the numbers in curly brackets in this protocol refer to SPIRIT checklist item numbers. The order of the items has been modified to group similar items (see http://www.equator-network.org/reporting-guidelines/spirit-2013-statement-defining-standard-protocol-items-for-clinical-trials/).

**Table:** 

Title {1}	VeSpAR trial: a randomized controlled trial comparing Vessel-Sparing Anastomotic Repair and transecting anastomotic repair in isolated short bulbar urethral strictures
Trial registration {2a and 2b}.	This trial is registered at clinicaltrials.gov (NCT03572348) and in the Belgian Clinical Trial Registry (B670201837335). Date of registry (dd/mm/yyyy): 28/06/2018 Register link: https://clinicaltrials.gov/ct2/show/NCT03572348?cond=vespar&draw=2&rank=1 Date first participant enrolled (dd/mm/yyyy): 26/09/2018
Protocol version {3}	Protocol version 1.2 Date (dd/mm/yyyy): 12/11/2019
Funding {4}	Not applicable as no funding was obtained for this trial.
Author details {5a}	Wesley Verla^1,2^, Marjan Waterloos^1^, Mieke Waterschoot^1^, Benjamin Van Parys^1^, Anne-Françoise Spinoit^1^, Nicolaas Lumen^1^ ^1^ Department of Urology, Ghent University Hospital, C. Heymanslaan 10, 9000 Ghent, Belgium. ^2^ Corresponding author: Wesley Verla > Email: wesley.verla@uzgent.be > Address: C. Heymanslaan 10, 9000 Ghent, Belgium > Tel.: (+32) 9 332 22 76 > Fax: (+32) 9 332 38 89 > ORCID: 0000-0001-5755-7078 Other email addresses: Marjan Waterloos: marjan.waterloos@hotmail.com Mieke Waterschoot: mieke.waterschoot@uzgent.be Benjamin Van Parys: benjamin.vanparys@uzgent.be Anne-Françoise Spinoit: anne-francoise.spinoit@uzgent.be Nicolaas Lumen: nicolaas.lumen@uzgent.be
Name and contact information for the trial sponsor {5b}	Ghent University Hospital Contact information: Clinical Trials Unit E-mail: HIRUZ.CTU@UZGENT.BE Tel.: +32 9 332 05 00
Role of sponsor {5c}	- Support in preparation of a correct and complete submission package for the Ethical Committee. - Performing on-site and remote monitoring according to International Council for Harmonization of Technical Requirements for Pharmaceuticals for Human Use – Good Clinical Practice (ICH-GCP) guidelines. - Trial monitoring in terms of safety reporting, annual progress reports and end of study reporting. - Providing a no-fault insurance.

## Introduction

### Background and rationale {6a}

Urethral stricture disease is a common urological condition in men. Although rigorous epidemiologic data are sparse, the existing papers report an incidence varying between 0.6 and 1.4% [1]. Urethral strictures can occur throughout the entire length of the urethra, but mainly involve the anterior urethra and in particular the bulbar segment [2].

The International Consultation on Urologic Diseases (ICUD) recommends anastomotic repair (AR) urethroplasty for isolated short bulbar urethral strictures [3]. This approach, in which the diseased bulbar urethral segment is excised and an end-to-end anastomosis is performed, is an optimal choice for strictures up to 3 cm and provides a composite success rate of 93.8% [3].

Traditionally, AR urethroplasty involved full thickness transection of the corpus spongiosum and the embedded urethral blood supply. However, it is only required to excise the narrow segment of the urethra and the surrounding spongiofibrosis, and therefore, a full thickness transection of the corpus spongiosum is in fact unnecessary. Against this background, Jordan et al. published an alternative, vessel-sparing technique in 2007 [4, 5], and since then, several urethroplasty centers have implemented this technique in their surgical repertoire [6–10]. It should be noted though that in some of these centers the vessel-sparing technique does not involve an active dissection and isolation of the bulbar arteries at the bulb of the corpus spongiosum, in contrast to Jordan et al., as this dissection is in fact unnecessary, time-consuming, and more traumatizing [6, 7].

Anyhow, the act of leaving the bulbar arteries intact in vessel-sparing anastomotic repair (vsAR) could offer certain functional benefits compared to the classic transecting anastomotic repair (tAR) technique. Preserving the bulbar arteries potentially reduces the risk of postoperative erectile dysfunction or glans ischemia, and it could be beneficial for subsequent interventions of the urethra; a free graft urethroplasty for instance, in which a rich vascular bed is essential; or the implantation of an artificial urinary sphincter, in which a well-sustained vascularization is imperative for success [4–7]. However, prospective studies comparing the functional outcome of both techniques with validated questionnaires are currently lacking, and thus, these potential benefits remain mere theoretical assumptions.

Be that as it may and potential functional benefits aside, vsAR should at least be able to provide a comparable (or non-inferior) surgical success rate as the transecting technique to be considered a truly valuable alternative. Promising short-term results have been described in single-arm observational studies and are in line with the reported composite success rate of the ICUD [3, 6–10]. However, direct comparisons between vsAR and tAR have only been done in retrospective studies which are strongly biased by the fact that patients who underwent vsAR had shorter follow-up than patients who underwent tAR [11, 12]. Moreover, since vsAR is a more novel technique, one could hypothesize that the performing surgeon is better trained and more experienced in the era of vsAR compared to the earlier in their surgical career, when tAR was the only AR technique.

Considering the above, the aim of this randomized controlled trial is to directly compare vsAR and tAR for both surgical and functional outcomes.

## Objectives {7}

The primary objective of this trial is to show that failure-free survival (FFS) after vsAR is non-inferior to tAR after 24 months of follow-up.

❖ Failure is defined as the inability to pass a 16-Fr flexible cystoscope through the reconstructed area without damaging the urethral mucosa.

❖ The non-inferiority limit, *d*, was set at 10%. This non-inferiority limit was chosen based on experts’ opinion, 5 patients’ opinion, and a round-the-table consensus discussion.

❖ The fact that failure after AR usually occurs within the first years after surgery serves as the rationale behind the follow-up interval of 24 months in this study [13].

Main research question, study hypothesis (Ha), and null hypothesis (H0) based on primary objective:

- Is the FFS after vsAR not inferior to the FFS after tAR in isolated short bulbar urethral strictures?
- Study hypothesis (Ha): The FFS after vsAR is not inferior to the FFS after tAR in isolated short bulbar urethral strictures.
- Null hypothesis (H0): The FFS after vsAR is inferior to the FFS after tAR in isolated short bulbar urethral strictures.

## Trial design {8}

This trial is a prospective, interventional, multi-center, single-blinded, 1:1 randomized, controlled, non-inferiority, phase II trial.

## Methods: participants, interventions, and outcomes

### Study setting {9}

This international multi-center trial will be deployed in the following centers (referral centers for management of urethral stricture disease):

1. Department of Urology, Ghent University Hospital, C. Heymanslaan 10, 9000 Ghent, Belgium
2. Department of Urology, Hospital San José Tecnológico de Monterrey, Universidad de Monterrey, Nuevo León, Mexico (Local PI & correspondent: Oscar Arturo Suárez Fernández de Lara)
3. Department of Urology, Hospital de Santa María, Universidad de Lisboa, Lisboa, Portugal (Local PI & correspondent: Francisco Martins)
4. Department of Urology, Hospital Italiano de Buenos Aires, Buenos Aires, Argentina (Local PI & correspondent: Carlos Giudice)
5. Department of Urology, Eastern Virginia Medical School, Norfolk, VA, USA (Local PI & correspondent: Ramon Virasoro)
6. Centro de Uretra Las Alamedas, Mexico (Local PI & correspondent: Erick Ramirez)
7. Department of Urology, SUNY Upstate Medical University, Syracuse, NY, USA (Local PI & correspondent: Dmitriy Nikolavsky)
8. Department of Urology, University Hospital Leuven, Leuven, Belgium (Local PI & correspondent: Steven Joniau)
9. Department of Urology, Hospital Univ. Marqués de Valdecilla, Santander, Spain (Local PI and correspondent: Félix Campos)
10. Department of Urology, Shanghai Jiao Tong University Affiliated Sixth People’s Hospital, 200233 Shanghai, China (Local PI and correspondent: Lujie Song)
11. Department of Urology, University College London Hospital, London, UK (Local PI and correspondent: Tamsin Greenwell)
12. Department of Urology, Shaare Zedek Medical Center, Jerusalem, Israel (Local PI and correspondent: Ofer Shenfeld)
13. Department of Urology, Beijing Jishuitan Hospital, Beijing, China (Local PI and correspondent: Jianwei Wang)
14. Department of Urology, University Hospital of Liège, Liège, Belgium (Local PI and correspondent: Maxime Sempels)

### Eligibility criteria {10}

Inclusion criteria for participants:

- Voluntarily signed written informed consent according to the rules of ICH-GCP (Declaration of Helsinki) and national regulations.
- Age ≥ 18 years.
- Male patient.
- Fit for operation, based on the surgeon’s expert opinion.
- Isolated short (≤ 3 cm) bulbar urethral stricture confirmed by imaging. Imaging includes at least retrograde urethrography (RUG). In case of equivocal findings, voiding cysto-urethrography (VCUG) and urethroscopy must be added.
- Patient is able and willing to comply with the postoperative protocol.

Exclusion criteria for participants:

- Absence of signed written informed consent.
- Age < 18 years.
- Female patients.
- Transgender patients.
- Patients unfit for operation.
- Concomitant urethral strictures at other urethral locations (penile urethra, membranous urethra, prostatic urethra, bladder neck).
- Urethral strictures > 3 cm.
- Urethral stricture(s) at other urethral locations (penile urethra, membranous urethra, prostatic urethra, bladder neck).
- Lichen sclerosus-related strictures.
- Strictures after failed hypospadias repair.
- Patients with neurogenic bladder.
- Shift of technique to augmented urethroplasty or any technique other than AR due to any circumstance. These patients will be logged, but will be excluded (post-hoc) from further analysis within this trial.
- History of pelvic radiation therapy.
- Active treatment to enhance erectile function (such as PDE5-inhibitors and intracavernous injections) at the moment of prescreening for inclusion in this trial.
- Any condition or situation, which, in the investigator’s opinion, puts the patient at significant risk, could confound the study results, or may interfere significantly with the patient’s participation in the study.
- Patient declares that it will be impossible for him to attend the follow-up consultations.

Eligibility criteria for participating centers:

Only urethral surgeons who state that they are sufficiently capable of performing both vsAR and tAR are allowed to participate in this trial.

### Who will take informed consent? {26a}

Patients meeting the in- and exclusion criteria of this trial will be thoroughly informed about this trial by the local principal investigator (PI) and will be asked if they are willing to participate. If so, the local PI will ask the patient to sign the written informed consent form. All informed consent forms will be stored in a locked file cabinet, unavailable to anyone except the local PI.

### Additional consent provisions for collection and use of participant data and biological specimens {26b}

Not applicable as no biological specimens will be collected as part of this trial.

## Interventions

### Explanation for the choice of comparators {6b}

The comparator, tAR, has been the gold standard technique for as long as anastomotic repairs exist [3]. In 2007, vsAR was invented as a less traumatic variant of the classic tAR, hoping to optimize functional outcome after this procedure without jeopardizing the excellent FFS rates after this type of surgery [4, 5].

### Intervention description {11a}

All patients will be asked to sign the written informed consent form at the beginning of the study, and once signed, they will be asked to fill in the preoperative questionnaire.

Preoperatively, all patients will be asked to deliver a urine sample for urinalysis and urine culture. Patients with evidence of a urinary tract infection will be treated with adequate antibiotics according to the participating center’s standard of care.

The same routine perioperative care will be applied to patients of both groups.

Surgical approach will be different for patients of different groups:

- Main steps in vsAR involve: midline perineal skin incision, cleavage of the bulbospongious muscle, circumferential dissection of the bulbar urethra, mobilization of the urethra by freeing it circumferentially from the penoscrotal angle up to the urogenital diaphragm, dorsal longitudinal urethrotomy over the strictured area, resection of the urethral stricture and surrounding spongiofibrosis, dorsal spatulation of the healthy urethral ends, transverse closure of the urethra over a catheter, spongioplasty, closure of the bulbospongious muscle, and closure of the wound in multiple layers [14].
- Main steps in tAR involve: midline perineal skin incision, cleavage of the bulbospongious muscle, circumferential dissection of the bulbar urethra, mobilization of the urethra by freeing it circumferentially from the penoscrotal angle up to the urogenital diaphragm, resecting the entire urethral segment (with its surrounding corpus spongiosum) that is strictured, spatulation of the healthy urethral ends, transverse closure of the urethra over a catheter, spongioplasty, closure of the bulbospongious muscle and closure of the wound in multiple layers.

As mentioned above, routine postoperative care will be provided to all patients and will be similar for patients of both groups.

A voiding cysto-urethrography (VCUG) will be performed 10–14 days after surgery, and in case of no or insignificant contrast leakage, the transurethral catheter will be removed. In case of significant contrast extravasation, the catheter will be maintained and imaging will be repeated 1 week later until no more significant contrast leakage can be identified. This short-term imaging will be similar for patients of both groups.

Follow-up moments will be held at 3, 12, and 24 months postoperatively and will involve urethroscopy, filling in the postoperative questionnaire and uroflowmetry. Follow-up will be similar for patients of both groups.

### Criteria for discontinuing or modifying allocated interventions {11b}

Conversion of vsAR to tAR for any reason is allowed upon the operating surgeon’s discretion. These patients will be considered part of the vsAR group in the intention-to-treat (ITT) analyses and will be considered part of the tAR group in the per-protocol (PP) analyses.

All shifts of technique towards augmented urethroplasty or any technique other than AR due to any circumstance will be logged, but these patients will be excluded (post-hoc) from further analysis within this trial.

### Strategies to improve adherence to interventions {11c}

No specific strategies to improve adherence to interventions are foreseen.

### Relevant concomitant care permitted or prohibited during the trial {11d}

No concomitant care will be prohibited during the trial.

### Provisions for post-trial care {30}

A no-fault insurance is foreseen for all trial participants.

### Outcomes {12}

Primary end-point

- To show that FFS after vsAR is non-inferior to tAR after 24 months of follow-up.
  ❖ Failure is defined as the inability to pass a 16-Fr flexible cystoscope through the reconstructed area without damaging the urethral mucosa. This anatomical definition of failure was chosen based on the fact that it has—to the best of our knowledge—the highest sensitivity and specificity to detect stricture recurrence after urethroplasty. In our opinion, this end-point leaves little to no room for interpretation, whereas a functional definition of failure (“need for re-intervention”) might be strongly influenced by the treating physician (e.g., physicians who personally favor technique A over B might be more reluctant to offer redo surgery to this group compared to patients treated with technique B, thus introducing the biased result that A would be better than B because fewer patients have to be re-operated during the follow-up period).
  ❖ The non-inferiority limit, *d*, was set at 10%. This non-inferiority limit was chosen based on experts’ opinion, 5 patients’ opinion and a round-the-table consensus discussion.
  ❖ The fact that failure after AR usually occurs within the first years after surgery serves as the rationale behind the follow-up interval of 24 months in this study [13].

Secondary end-points

- To analyze change in erectile function and differences in change of erectile function between vsAR and tAR.
  ❖ The International Index of Erectile Function (IIEF-5) questionnaire* will be administered to participants preoperatively and after 3, 12, and 24 months of follow-up.
  ❖ The IIEF-5 questionnaire provides a summative score between 5 and 25 and should be interpreted as follows: 22–25 = no erectile dysfunction; 17–21 = mild erectile dysfunction; 12–16 = mild to moderate erectile dysfunction; 8–11 = moderate erectile dysfunction; 5–7 = severe erectile dysfunction.
  ❖ De novo postoperative worsening of erectile function will be defined as a decrease of ≥ 5 points on the postoperative IIEF-5 questionnaire compared to the preoperative IIEF-5 questionnaire. This definition was chosen based on experts’ opinion, taking into account the lack of studies describing the ideal cut-off value to delineate “de novo erectile dysfunction” for this 5-item questionnaire [12].
  To analyze change in ejaculatory function and differences in change of ejaculatory function between vsAR and tAR.
  ❖ The Male Sexual Health Questionnaire—Ejaculatory Dysfunction short form (MSHQ-EjD short form) questionnaire** will be administered to participants preoperatively and after 3, 12, and 24 months of follow-up.
  ❖ The MSHQ-EjD short form questionnaire, consisting of 4 questions, provides a summative score between 1 and 15 (question 1–3) and a bother/satisfaction score between 0 and 5 (question 4).
  To analyze change in lower urinary tract symptoms (LUTS) and differences in change of LUTS between vsAR and tAR.
  ❖ The International Consultation on Incontinence Questionnaire—Male Lower Urinary Tract Symptoms module (ICIQ-MLUTS module) questionnaire** will be administered to participants preoperatively and after 3, 12, and 24 months of follow-up.
  ❖ The ICIQ-MLUTS module, consisting of 7 questions, provides a summative score between 0 (asymptomatic) and 24 (most symptomatic) (question 1–6) and a bother score between 0 (not bothersome) and 3 (very bothersome) (question 7).
  ❖ Remark: The 7 questions of the ICIQ-MLUTS module questionnaire are only a subset of the entire ICIQ-MLUTS questionnaire. These 7 questions were chosen because of their relevance in a setting of urethral stricture disease and were validated in English, Dutch, and other languages (cfr. infra).
  ❖ Peeling’s voiding picture** will be administered to participants preoperatively and after 3, 12, and 24 months of follow-up.
  ❖ Peeling’s voiding picture provides a score between 1 and 4.
- To analyze change in urinary incontinence and differences in change of urinary incontinence between vsAR and tAR.
  ❖ The International Consultation on Incontinence Questionnaire—Urinary Incontinence short form (ICIQ-UI short form) questionnaire will be administered to participants preoperatively and after 3, 12, and 24 months of follow-up.
  ❖ The ICIQ-UI short form questionnaire, consisting of 3 scoring questions and 1 self-diagnostic question, provides a summative score between 0 (asymptomatic) and 21 (most symptomatic).
- To analyze change in quality of life and differences in change of quality of life between vsAR and tAR.
  ❖ The EQ-5D-3L questionnaire** will be administered to participants preoperatively and after 3, 12, and 24 months of follow-up.
  ❖ The EQ-5D-3L module, consisting of 5 questions, provides a 5-digit score in which every digit varies between 1, 2, and 3.
  ❖ The EQ—Visual Analogue Scale (EQ-VAS)** will be administered to participants preoperatively and after 3, 12, and 24 months of follow-up.
  ❖ The EQ-VAS provides a scale between 0 and 100.
- To analyze patient satisfaction and differences in patients’ satisfaction between vsAR and tAR.
  ❖ Two specific questions regarding patient satisfaction will be asked at 3, 12, and 24 months.
- To analyze change in maximum flow rate (Qmax) and differences in Qmax between vsAR and tAR.
  ❖ Uroflowmetry will be performed preoperatively and after 3, 12 and 24 months of follow-up.
- To analyze postoperative (< 90 days) complication rate and differences in postoperative (< 90 days) complication rate between vsAR and tAR.
  ❖ Complications within the first 90 postoperative days will be categorized according to the Clavien-Dindo classification system [15, 16].

* The IIEF-5 questionnaire is a validated tool to assess erectile function and can be used in patients undergoing urethroplasty [17, 18].

** These subsets of questions were validated in English and in Dutch in a setting of patients with urethral strictures undergoing urethroplasty [19, 20].

### Participant timeline {13}

**Fig. 1:**
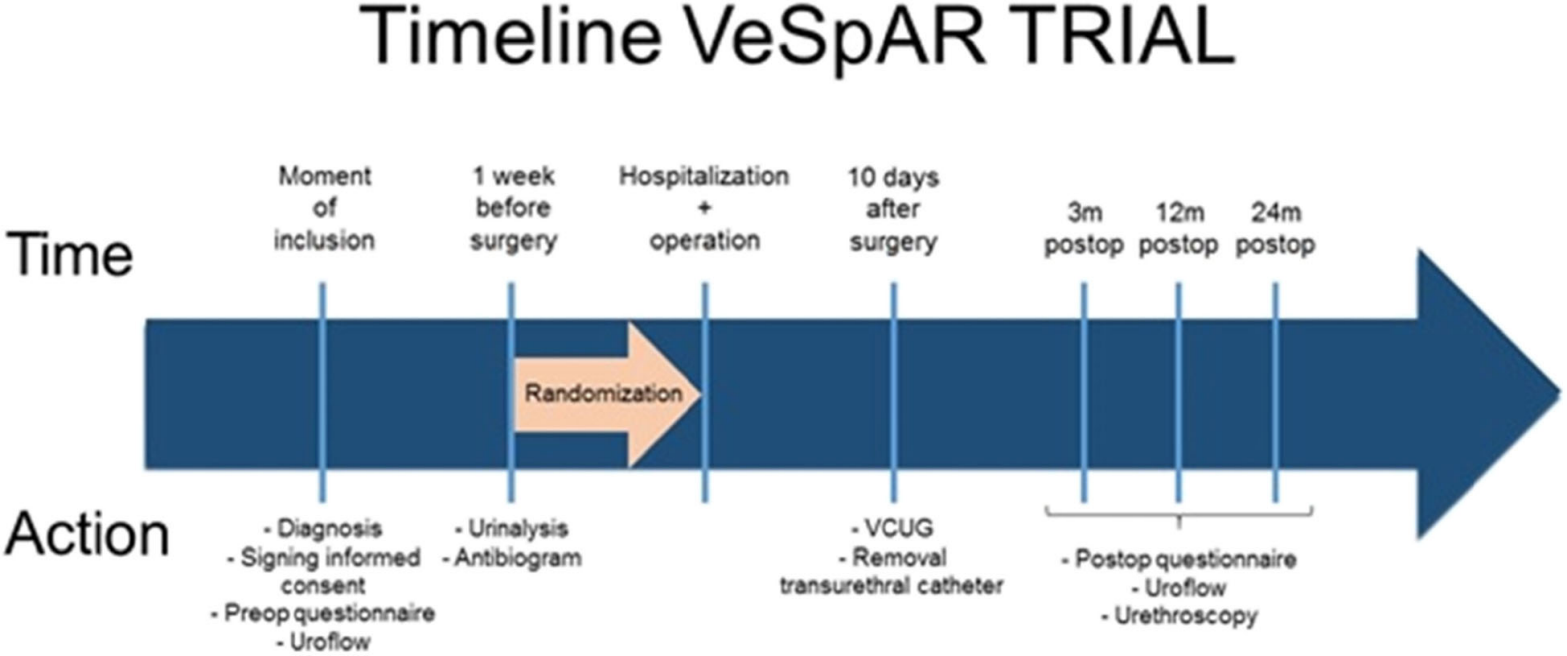
Timeline VeSpAR trial. VCUG, voiding cysto-urethrography

Timeline VeSpAR trial is shown in Fig. 1.

### Sample size {14}

Power analysis and sample size calculation for this non-inferiority trial were performed using an online software package:

Sealed Envelope Ltd. 2012. Power calculator for binary outcome non-inferiority trial. [Online] Available from: https://www.sealedenvelope.com/power/binary-noninferior/ [Accessed Mon Apr 162,018].

Different scenarios were simulated, of which the results are discussed below. In all scenarios, 10% was considered the non-inferiority limit, *d*, as mentioned above.

- Scenario 1: power 0.80; estimated success in control group (tAR): 95.4%; estimated success in experimental group (vsAR): 95.3%; for different one-sided alphas:

❖ Alpha 0.025: 72 patients per arm

❖ Alpha 0.05: 56 patients per arm

❖ Alpha 0.10: 41 patients per arm

- Scenario 2: power 0.80; estimated success in control group (tAR): 95.4%; estimated success in experimental group (vsAR): 95.4%; for different one-sided alphas:

❖ Alpha 0.025: 69 patients per arm

❖ Alpha 0.05: 55 patients per arm

❖ Alpha 0.10: 40 patients per arm

- Scenario 3: power 0.80; estimated success in control group (tAR): 97.4%; estimated success in experimental group (vsAR): 93%; for different one-sided alphas:

❖ Alpha 0.025: 227 patients per arm

❖ Alpha 0.05: 179 patients per arm

❖ Alpha 0.10: 131 patients per arm

The numbers that were put in the above simulations are based on an in-home analysis of patients who underwent anastomotic repair at Ghent University Hospital between 2000 and 2017. Herein the FFS at 24 months for vsAR and tAR were 95.3% (standard deviation (SD), 2.3%) and 95.4% (SD, 2.0%) respectively. These exact results are represented in scenario 1. Scenario 2 represents a situation in which the surgical outcome would be exactly the same for vsAR and tAR. Scenario 3 represents a less favorable situation in which vsAR has a 24 m-FFS of 93% (95.3% − SD) and tAR has a 24 m-FFS of 97.4 (95.4% + SD).

In every scenario, the power was set at 0.80, according to the guidelines for designing a phase II trial [21]. The results for different alphas are displayed above. Usually, a non-inferiority trial demands a significance level, alpha, of 0.025 as these trials only look at one boundary of the classic two-sided 95% confidence interval (CI) [22]. However, these conditions often lead to (sometimes unreasonably) high sample size calculations requiring excessive financing and recruitment. As a solution, researchers often design a phase II trial that allows for more coincidence and thus a trial with a higher significance level, alpha. Rubinstein et al. reported alpha values of 0.10 and 0.20 to be allowed in a phase II trial [21]. Considering the fact that a non-inferiority trial relies on one-sided testing, we believe a one-sided significance level, alpha, of 0.10 is allowed and most feasible.

Considering all of the above, a scenario in which the power is set at 0.80, the one-sided significance level, alpha, is set at 0.10, the estimated success in control group is set at 95.4%, and the estimated success in experimental group is set at 95.3% was found most realistic and feasible. Considering this and accounting for a realistic drop-out of 20% (experience based), it was estimated that each arm has to consist of 50 patients, resulting in a total sample size of 100 patients.

### Recruitment {15}

**Fig. 2:**
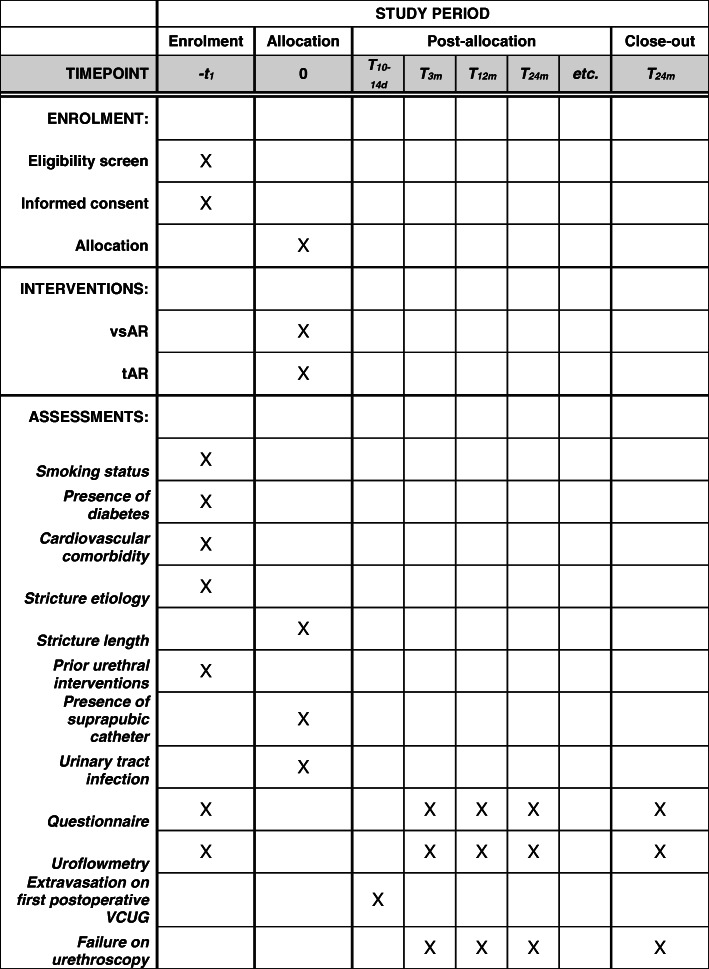
SPIRIT figure for enrolment, interventions, and assessments. vsAR, vessel-sparing anastomotic repair; tAR, transecting anastomotic repair; VCUG, voiding cysto-urethrography

All patients meeting the inclusion criteria of the trial and presenting at one of the participating centers will be informed about the trial and will be asked to participate (Fig. 2).

Strategies for achieving adequate participant enrolment in this trial include:

- Repetitive review of the recruitment status (overall and per participating center) every 3 months.
- Informing local PIs about their recruitment status and how this relates to other study sites.

The recruitment period is estimated to end in January 2021.

## Assignment of interventions: allocation

### Sequence generation {16a}

Random allocation of patients to one of both treatment arms will occur through a computer-based random sequence generator with permuted blocks of variable size. Randomization will be stratified by participating center.

### Concealment mechanism {16b}

Random allocation of patients to one of both treatment arms will be done by a third party who is further not involved in any process of the trial. When a participating center other than Ghent University Hospital wants to allocate a patient to one of both treatment arms, the local PI will contact the third party for randomization within 1 week before the planned operation (by central telephone number or e-mail address) and will ask to which treatment arm “patient X who is going to be operated on dd/mm/yyyy” is allocated. Note that there will be no communication of names, initials, or birth dates of patients; only pseudonymized data will be transferred. After receival of this request for randomization of a patient, the third party for randomization will assign a unique trial identifier to the patient at hand and will respond to which treatment arm this patient has been allocated.

As such, the randomization process is concealed for the treating surgeon up until 1 week before surgery. Since these two different techniques may entail different operating room preparations (different retractors, different sutures, etc.), randomization concealment for the treating surgeon is lifted 1 week preoperatively for logistic considerations.

### Implementation {16c}

Random allocation of patients to one of the treatment arms will be done by a third party who is not involved in any of the trial procedures.

Patients will be enrolled in the trial by the local PIs, and after communicating the pseudonymized data of a new trial participant, the third party for randomization will assign the participant at hand to one of both treatment arms (cfr. supra).

## Assignment of interventions: blinding

### Who will be blinded {17a}

Trial participants will be blinded and, as mentioned above, randomization of patients will be concealed for the treating surgeon up until 1 week preoperatively.

Blinding of outcome assessors and data analysts was considered, but we decided against this due to practical considerations.

### Procedure for unblinding if needed {17b}

Upon patient’s request, unblinding is allowed after termination of the trial.

In case of any unforeseen medical emergency requiring unblinding of the trial participant, unblinding will be allowed as well.

Unblinding will occur through face-to-face contact between the trial participant and the local PI who may then reveal the exact surgical technique that was performed.

## Data collection and management

### Plans for assessment and collection of outcomes {18a}

FFS after 3, 12, and 24 months will be assessed by urethroscopy using a flexible 16-Fr cystoscope. Not being able to pass this flexible cystoscope past the area of reconstruction will be logged as a failure.

Changes in functional outcome 3, 12, and 24 months after the operation will be assessed with validated questionnaires, as mentioned above. The scores on these questionnaires will be compared with the baseline questionnaire every patient has to fill in preoperatively. A short list of these questionnaires can be found hereafter:

- Change in erectile function: IIEF-5 questionnaire
- Change in ejaculatory function: MSHQ-EjD short form questionnaire
- Change in LUTS: ICIQ-MLUTS module and Peeling’s voiding picture
- Change in urinary incontinence: ICIQ-UI short form questionnaire
- Change in quality of life: EQ-5D-3L questionnaire and EQ-VAS
- Postoperative patient satisfaction: 2 separate questions.

Change in Qmax will be assessed by uroflowmetry at 3, 12, and 24 months postoperatively and will be compared to the preoperative Qmax.

Postoperative complications will be assessed by the treating surgeon through thorough history taking and physical examination at the 3-month follow-up moment. Surgical complications will be logged and categorized according to the Clavien-Dindo classification system [15, 16].

Every local PI of every participating center will receive an electronic document stipulating how to assess the aforementioned outcome variables and how to enter these data in the online data registry (cr. infra). The follow-up of patients within this trial is comparable to the follow-up of urethroplasty patients outside the context of a clinical trial, and therefore, no specific training for outcome assessors is foreseen.

### Plans to promote participant retention and complete follow-up {18b}

Participant retention will be promoted by scheduling every foreseen follow-up moment (10 days, 3 months, 12 months, and 24 months postoperatively) in advance, ideally at the moment of inclusion or at the moment of discharge from the hospital. If patients do not show up at the foreseen follow-up moment and do not reschedule, they will be invited for a new appointment by telephone or e-mail.

### Data management {19}

The local PIs of the participating centers will enter all required data individually in a firewall protected online data registry which complies with the guidelines of good clinical practice. Access to this online data registry is only possible upon invitation by the central PI or the trial coordinator and by use of a unique, center-specific password. All data will be stored in a central IBM SPSS database which is protected by an institutional firewall system. All data, entered and stored, will be pseudonymized to comply with current privacy regulations. Local PIs will have access to the data of their own patients enrolled in this trial. The central PI and the trial coordinator will have access to all data. Before the start of recruitment, every local PI of every participating center will receive an electronic document including a list of variables that will be collected and thorough instructions on how to enter data. A manual bimonthly data check will be performed by the trial coordinator to optimize data quality.

### Confidentiality {27}

Data entry and communication about trial participants will be pseudonymized to guarantee confidentiality, before, during, and after the trial. Pseudonymized patient data will only be accessible for members of the research team.

### Plans for collection, laboratory evaluation, and storage of biological specimens for genetic or molecular analysis in this trial/future use {33}

Not applicable as no biological specimens will be collected as part of this trial.

## Statistical methods

### Statistical methods for primary and secondary outcomes {20a}

ITT analyses will be used for all end-points.

#### Analysis of the primary end-point

Kaplan-Meier curves for FFS will be plotted with the corresponding log-log 80% CIs for vsAR and tAR.

Non-inferiority of vsAR versus tAR in terms of FFS at 24 months will be concluded when the lower limit of the 80% CI for the difference in FFS probability at 24 months does not exceed − 10%. Note that this analysis does not take into account the nesting of patients within centers, which may affect the estimated standard errors.

To take into account the clustering of patients within centers, a marginal Cox proportional hazards model can be fitted (using the cluster function from the “survival” package in R software) which will give a robust estimation of the variance. The lower limit of the 80% CI for the hazard ratio can be transformed into a limit of survival difference at 24 months, assuming an exponential distribution of failure-free survival times [*S*(*t*) = exp.{−*λt*}].

#### Analysis of the secondary end-points

Postoperative changes in erectile function, ejaculatory function, LUTS, urinary incontinence, and quality of life will be analyzed by comparing the scores of the postoperative questionnaires with the scores of the preoperative questionnaires. For every participant, the 3-, 12-, and 24-month follow-up questionnaire scores will be compared to the respective preoperative questionnaire scores. Differences in scores will be logged for the 3-, 12-, and 24-month follow-up moment, and for each of these time-points, the median (interquartile range (IQR)) change in scores will be assessed for vsAR and tAR. Median (IQR) change in scores will be compared between vsAR and tAR using a two-sided Mann-Whitney *U* test at the 20% significance level.

As regards de novo postoperative worsening of erectile function, defined as a decrease of ≥ 5 points on the postoperative IIEF-5 questionnaire compared to the preoperative IIEF-5 questionnaire, a two-sided Fisher’s exact test at the 20% significance level will be used to compare vsAR and tAR at the 3-, 12-, and 24-month follow-up moment.

Postoperative patient satisfaction will be assessed at the 3-, 12-, and 24-month follow-up moment. For each time-point, the proportion of satisfied patients will be compared between vsAR and tAR using a two-sided Fisher’s exact test at the 20% significance level.

Postoperative change in Qmax will be compared between vsAR and tAR at the 3-, 12-, and 24-month follow-up moment using a two-sided Mann-Whitney *U* test at the 20% significance level.

Postoperative complications will be compared between vsAR and tAR using a two-sided Fisher-Freeman-Halton test at the 20% significance level.

Note that for all secondary end-points, participants will be censored at the moment they undergo a subsequent urethral treatment (dilation, urethrotomy, urethroplasty, or urinary diversion).

### Interim analyses {21b}

A formal interim analysis for safety is planned when the 3-month follow-up data of the first 50 randomized patients are obtained. This analysis will be performed by the independent third party for safety and the trial coordinator. The following stop criteria have been predefined:

- 3 m-FFS of vsAR and its two-sided 95% CI are entirely beneath the 3 m-FFS of tAR – 10%.
- > 30% difference between vsAR and tAR in de novo postoperative worsening of erectile function, defined as a decrease of ≥ 5 points on the postoperative IIEF-5 questionnaire compared to the preoperative IIEF-5 questionnaire.

If one of the stop criteria is reached, the independent third party for safety will discuss this with the central PI and the central Ethical Committee, and a decision about prematurely stopping the trial will be formulated and communicated to all parties involved in the trial.

### Methods for additional analyses (e.g., subgroup analyses) {20b}

Interaction tests for FFS were considered for traumatic versus non-traumatic stricture etiology and for primary versus secondary procedures. However, the rule of thumb says that at least 10 failures are needed per degree of freedom included in the model. Allowing for a modification of the effect of procedure by stricture etiology for instance would imply adding 3 degrees of freedom to the model (technique, center, and stricture etiology). This would lead to overfitting, which means the obtained results might not be reproducible in other samples. We will refrain from generalizing our findings from these (over)complex models to the larger population of patients and stick to purely descriptive results when considering more than one explanatory covariate.

The PP analysis of de novo postoperative worsening of erectile function after vsAR and tAR will be performed as well (two-sided Fisher’s exact test at the 20% significance level) to explore the effect of full-thickness transection on postoperative erectile function.

### Methods in analysis to handle protocol non-adherence and any statistical methods to handle missing data {20c}

Participant retention, protocol adherence, and minimization of missing data will be promoted by scheduling every foreseen follow-up moment (10 days, 3 months, 12 months, and 24 months postoperatively) in advance, ideally at the moment of inclusion or at the moment of discharge from the hospital. If patients do not show up at the foreseen follow-up moment and do not reschedule, they will be invited for a new appointment by telephone or e-mail.

In case of drop-out, patients will be contacted by the local PI and the reason for drop-out will be logged qualitatively. In case of no further contact with the participant at hand, “unknown reason for drop-out” will be logged. These participants will be censored at the moment of latest follow-up.

In case of protocol non-adherence, the reason for this will be logged qualitatively by the local PI.

Missing or incorrect data will be detected by software programs and will be reported transparently in the publication of trial results.

### Plans to give access to the full protocol, participant level-data and statistical code {31c}

The datasets generated and/or analyzed during the current study are not publicly available due to the European Union General Data Protection Regulation, but are available from the corresponding author on reasonable request.

## Oversight and monitoring

### Composition of the coordinating center and trial steering committee {5d}

Ghent University Hospital will be the coordinating center, and the trial steering committee will be composed as follows:

- Central PI = prof. dr. Nicolaas Lumen
- Trial coordinator = dr. Wesley Verla
- Back-up trial coordinator = dr. Marjan Waterloos
- Central subinvestigators = dr. Wesley Verla, dr. Marjan Waterloos, dr. Mieke Waterschoot, dr. Benjamin Van Parys, dr. Anne-Françoise Spinoit
- Independent third party for random allocation of trial participants to one of both treatment arms.
- Independent third party for data monitoring and protection of patient safety by analyzing the harm of both treatment arms.
- An institutional biostatistician.
- An institutional data protection officer will protect every process of data management and will verify whether each and every step in this process is according to current privacy regulations.
- The clinical trials unit of Ghent University Hospital’s ‘Health, Innovation and Research’ department will oversee trial progress and steer where necessary.

### Composition of the data monitoring committee, its role and reporting structure {21a}

There will be no formal data monitoring committee independent from the sponsor or the trial steering committee. Patient safety will be protected by an independent third party who will bimonthly assess the harm of both treatment arms. This independent third party for safety has no conflict of interest with the sponsor of this trial. Stop criteria have been predefined by the trial steering committee (cfr. supra). If one of the stop criteria is reached, the independent third party for safety will discuss this with the central PI and the central Ethical Committee and a decision about prematurely stopping the trial will be formulated and communicated to all parties involved in the trial. A formal interim analysis for safety is planned when the 3-month follow-up data of the first 50 randomized patients are obtained.

### Adverse event reporting and harms {22}

Adverse events and postoperative complications will be assessed by the treating surgeon through thorough history taking, physical examination, or by any means necessary. If present, these adverse events and postoperative complications will be logged and categorized according to the Clavien-Dindo classification system [15, 16]. Treatment of adverse events and postoperative complications will take place according to the best standard of care of the participating center at hand.

### Frequency and plans for auditing trial conduct {23}

A yearly audit will be organized by the clinical trials unit of Ghent University Hospital’s “Health, Innovation and Research” department.

### Plans for communicating important protocol amendments to relevant parties (e.g., trial participants, ethical committees) {25}

All important protocol amendments will be communicated by e-mail to the central Ethical Committee and to all local PIs, who are in turn responsible to communicate this to their own local Ethical Committee.

## Dissemination plans {31a}

Trial results will be published in the format of an original article which will be submitted for peer review and publication in an A1 journal. Besides, an abstract of the trial’s results will be submitted for presentation at national and international scientific meetings. Trial results will be published irrespective of the direction or nature of the findings.

## Discussion

This study will provide evidence about whether vsAR yields non-inferior surgical results compared to tAR or not. This question has been subject to debate for over a decade, and yet, only retrospective research is available on this topic. To the best of our knowledge, this trial is the first randomized controlled trial to address the question at hand.

Beside the comparison of surgical outcome after both techniques, this trial will also investigate differences in change of functional outcome parameters between both techniques. This will further elucidate the true value of bulbar artery preservation and either refute or confirm the advocated theories about functional benefits.

Depending on the trial results, this phase II trial may be the basis for a larger phase III trial with more statistical power and a lower alpha value.

## Trial status

The first patient was included on September 29, 2018. All trial participants are scheduled to be enrolled by January 2021. The outlined protocol is protocol version 1.2 (November 12, 2019).

## Abbreviations

AR: Anastomotic repair
CI: Confidence interval
FFS: Failure-free survival
ICUD: International Consultation on Urologic Diseases
ICIQ-MLUTS: International Consultation on Incontinence Questionnaire—Male Lower Urinary Tract Symptoms module
ICIQ-UI: International Consultation on Incontinence Questionnaire—Urinary Incontinence
ICH-GCP: International Council for Harmonization of Technical Requirements for Pharmaceuticals for Human Use—Good Clinical Practice
IIEF: International Index of Erectile Function
IQR: Interquartile range
ITT: Intention-to-treat
LUTS: Lower urinary tract symptoms
MSHQ-EjD: Male Sexual Health Questionnaire—Ejaculatory Dysfunction
PI: Principal investigator
PP: Per-protocol
Qmax: Maximum flow rate
RUG: Retrograde urethrography
SD: Standard deviation
tAR: Transecting anastomotic repair
VAS: Visual Analogue Scale
VCUG: Voiding cysto-urethrography
VeSpAR: Vessel-sparing anastomotic repair
vsAR: Vessel-sparing anastomotic repair
